# Real‐World Prevalence, Treatment Patterns, and Outcomes for Patients With HER2 (*ERBB2*)‐Mutant Metastatic Non‐Small Cell Lung Cancer, From a US‐Based Clinico‐Genomic Database

**DOI:** 10.1002/cam4.70272

**Published:** 2024-12-18

**Authors:** Sarah Waliany, Misako Nagasaka, Leah Park, Clara Lam, Zoe Jiang, Feng Lin, Joel W. Neal

**Affiliations:** ^1^ Department of Medicine Massachusetts General Hospital Cancer Center Boston Massachusetts USA; ^2^ University of California Irvine School of Medicine Orange California USA; ^3^ AstraZeneca Gaithersburg Maryland USA; ^4^ Daiichi Sankyo Basking Ridge New Jersey USA; ^5^ Department of Medicine Stanford University Stanford California USA

**Keywords:** advanced non–small cell lung cancer, ERBB2 mutation, HER2 mutation, targeted therapy

## Abstract

**Objectives:**

Targeted therapies have been shown to improve outcomes in metastatic non‐small cell lung cancer (mNSCLC) with driver mutations. We evaluated the real‐world prevalence of human epidermal growth factor receptor 2 (HER2; *ERBB2*) tumor gene mutations among patients with mNSCLC and described historical treatments and outcomes in patients with *HER2*‐mutant mNSCLC, during a period when there was no approved targeted therapy for *HER2*‐mutant mNSCLC.

**Materials and Methods:**

This retrospective observational study used a US nationwide de‐identified NSCLC clinico‐genomic database. Eligible patients were adults diagnosed with *HER2*‐mutant mNSCLC from January 2014 to July 2021 without co‐occuring epidermal growth factor receptor (*EGFR*) tumor mutations. Descriptive statistics were used to summarize prevalence, baseline characteristics and treatment patterns. Clinical outcomes were estimated with Kaplan–Meier analyses.

**Results:**

Among 9206 patients with mNSCLC, 164 (1.78%) met the eligibility criteria (mean age: 67.3 years, 63.4% White, 56.7% female, and 53.0% with a smoking history). 132/164 (80.5%) had at least one line of treatment. Platinum‐based chemotherapy (45.5%) and immune checkpoint inhibitor (ICI) with chemotherapy (28.0%) were the most frequently used first‐line treatments. The median (95% confidence interval [CI]) real‐world (rw) progression‐free survival in first‐line was 5.5 (4.8, 6.2) months and 3.0 (2.3, 4.2) months in second‐line. The median rw overall survival in first‐line was 13.2 (10.6, 18.4) months and 8.2 (6.6, 13.2) months in second‐line.

**Conclusion:**

During this study period, the most common regimens were platinum‐based chemotherapy with or without ICI in first and second line, and median rwOS was 13.2 and 8.2 months, respectively. These results indicate the need for more effective targeted therapies in this patient population.

## Abstract 2

Prior to the approval of targeted therapy for HER2‐mutant mNSCLC, HER2‐targeted therapies were mostly used in the later lines of treatments. Most patients received platinum‐based chemotherapy ± immunotherapy in first‐ and second‐line with less than 14 months and 10 months of median real‐world overall survival from first‐ and second‐line treatment initiation, respectively.

## 1 Introduction

Non‐small cell lung cancer (NSCLC) represents 81% of lung cancers [1]. The 5‐year survival rate is estimated to be 25% for all patients with NSCLC and 7% for patients with metastatic NSCLC (mNSCLC), though some tumors may be particularly sensitive to immunotherapy or targeted therapy and have much better outcomes [2]. Advances in the understanding of molecular alterations of tumors and discovery of actionable oncogenic driver mutations in NSCLC have led to the development of targeted therapies and transformed lung cancer treatment strategies. The detection of mutations such as epidermal growth factor receptor (*EGFR*) mutations, anaplastic lymphoma kinase (*ALK*) gene rearrangements, and c‐ROS oncogene 1 (*ROS1*) gene rearrangements has led to a paradigm shift in the treatment of mNSCLC from chemotherapy to targeted therapy. Therefore, routine testing for actionable mutations has become an important part of patient care in mNSCLC [3, 4]. Targeted therapies for the treatment of patients with mNSCLC have improved response rates, progression‐free survival (PFS), and overall survival (OS) in some clinical trials and real‐world studies [5, 6, 7, 8].

Human epidermal growth factor receptor 2 (HER2; ERBB2) as a tyrosine kinase receptor plays an important role in cell growth and development, and is expressed/overexpressed in various types of solid tumors including breast, gastric, ovarian, colon, and lung [9, 10, 11, 12]. It is reported that the co‐occurrence of *HER2* mutations with other actionable oncogenic drivers is low [13, 14]. Of the activating HER2 mechanisms, *HER2* gene mutations have been recognized as actionable biomarkers in mNSCLC [15]. *HER2* mutations occur predominantly within the tyrosine kinase domain of exon 20 [16, 17, 18, 19, 20, 21] but could also occur in extracellular and transmembrane domains [16]. From a prognostic perspective, patients with *HER2*‐mutant NSCLC tend to have poor OS [13]. Patients with *HER2*‐mutant mNSCLC have been shown to have a shorter median (m)OS compared with *HER2* wild‐type disease or those who harbor other oncogenic driver mutations including *EGFR* mutations or *ALK* rearrangements [13, 17].

Before the approval of trastuzumab deruxtecan (T‐DXd) in August 2022 by the US Food and Drug Administration (FDA) in previously treated mNSCLC, patients with mNSCLC with *HER2* mutations had been treated generally with chemotherapies or immune checkpoint inhibitors (ICIs) in first‐line treatments similar to treatment strategies in NSCLC with non‐actionable biomarkers [18, 19, 20, 21]. Owing to poor clinical outcomes, HER2‐directed therapies such as trastuzumab‐based regimens had not been commonly used in NSCLC until the approval of T‐DXd [22, 23, 24]. Compared to breast and gastric cancer studies where HER2‐directed treatments have demonstrated significant improvement in OS, clinical trials evaluating trastuzumab plus chemotherapy have not shown notable clinical benefits in patients with *HER2*‐positive NSCLC [25, 26, 27, 28, 29, 30, 31]. Antibody‐drug conjugates (ADCs) and HER2 tyrosine kinase inhibitors (TKIs) are newer targeted treatment options in this patient population. As the only HER2‐directed therapy approved in previously treated *HER2*‐mutant mNSCLC, T‐DXd (6.4 mg/kg) has demonstrated a mPFS of 8.2 (95% confidence interval [CI] 6.0, 11.9) months and a mOS of 17.8 (95% CI 13.8, 22.1) months in the Phase 2 DESTINY‐Lung01 trial [15, 32]. The US FDA approval was then based on the DESTINY‐Lung02 trial (5.4 mg/kg dose), which showed a confirmed overall response rate (ORR) of 57.7% (95% CI 43.2, 71.3) with a mPFS of 9.9 months (95% CI 7.4, not estimable [NE]) and a mOS of 19.5 months (95% CI 13.6, NE) [32, 33]. Studies evaluating the ADC trastuzumab emtansine (T‐DM1) or HER2 TKIs including pyrotinib and poziotinib have shown conflicting results [15]. With emerging targeted treatments in *HER2*‐mutant mNSCLC, we aimed to describe historical treatment patterns and clinical outcomes in a real‐world (rw) setting in this patient population before T‐DXd approval.

## 2 Materials and Methods

### 2.1 Data Sources

This retrospective observational study used the US‐based de‐identified Flatiron Health‐Foundation Medicine Inc. NSCLC Clinico‐Genomic Database (FH‐FMI CGDB). These data come from ~280 US cancer clinics (~800 sites of care). The FH dataset is a nationwide, longitudinal, and geographically diverse database covering electronic health record (EHR) data. Retrospective clinical data were derived from EHR data, consisting of patient‐level structured and unstructured data, curated via technology‐enabled abstraction, and were linked to genomic data derived from FMI comprehensive genomic profiling (CGP) tests in the FH‐FMI CGDB by de‐identified, deterministic matching. Rule‐based lines of therapy were defined by oncology clinicians. Biomarker status for *HER2* mutations as well as other biomarkers was available from the FH‐FMI CGDB based on next‐generation sequencing (NGS) of tissue. Genomic alterations were identified via CGP of > 300 cancer‐related genes on FMI's NGS test (using FMI's sequencing platform). The deidentified data were subject to obligations to prevent re‐identification and protect patient confidentiality.

### 2.2 Patient Population

**FIGURE 1:**
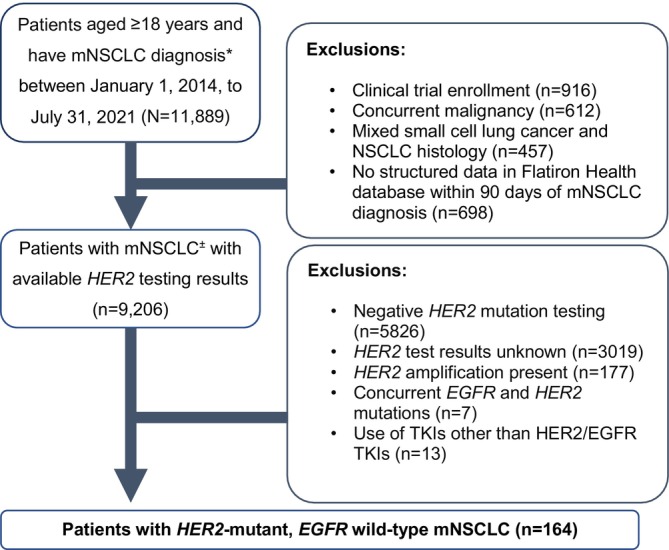
Patient selection diagram. *Patients had at least one diagnosis of lung cancer based on ICD‐9 or ICD‐10 codes (162.x, or C34.xx). ^±^Patients had to have an initial diagnosis of Stage IV NSCLC or have progressed to mNSCLC from an earlier stage, which was identified using a secondary tumor diagnosis code based on ICD‐9 or ICD‐10 codes (196.xx–198.xx or C77.xx, C79.xx, C7B.xx) recorded after the initial lung cancer diagnosis date and during the sample selection period, a record of tumor progression to Stage IV for patients whose initial diagnosis was early stage, or initiation of first‐line treatment for mNSCLC. EGFR, epidermal growth factor receptor; HER2, human epidermal growth factor receptor 2; ICD‐9, International Classification of Diseases, Ninth Revision; ICD‐10, International Classification of Diseases, Tenth Revision; mNSCLC, metastatic non‐small cell lung cancer; TKI, tyrosine kinase inhibitor.

The study population eligible for prevalence estimation was US patients aged ≥ 18 years diagnosed with mNSCLC from January 1, 2014 to July 31, 2021, and who had tumor DNA NGS results (Table S1). Patients were excluded if they had small cell histology or use of SCLC treatments, participation in clinical trials, concurrent malignancy, or absence of clinical data (e.g., physician visits, lab tests, non‐canceled drug orders) within 90 days of the first mNSCLC diagnosis. Patients whose tumors had concurrent *EGFR* tumor mutations or that used EGFR‐specific TKIs (other than afatinib) were also excluded, because the prior use of EGFR TKIs is a signal of EGFR‐mutant tumors with HER2 acquired resistance to EGFR inhibitors. Patients were followed until earliest of last follow‐up visit, death, or end of study (December 31, 2021). For other study outcomes including treatment patterns and clinical outcomes, we included patients with *HER2*‐mutant, *EGFR* wild‐type mNSCLC who met eligibility criteria. HER2 gene mutations were defined as known pathogenic or likely pathogenic variants of HER2 identified in the FH‐FMI CGDB. Detailed inclusion and exclusion criteria are shown in Figure 1. The index date was the initial diagnosis date of mNSCLC.

### 2.3 Study Measures

Patient characteristics were measured at baseline (defined as 6 months before the index date). These included demographic characteristics including smoking status and clinical characteristics such as sites of metastasis, tumor histology, *HER2* tumor mutation status, and Eastern Cooperative Oncology Group (ECOG) performance status.

Treatment patterns were assessed descriptively up to three lines of treatment after mNSCLC diagnosis. A line of treatment included all systemic antineoplastic treatments given within 28 days after the initiation of the line of treatment (Table S1). The end of line of treatment was defined as the earliest date of last visit, the last administration of treatment, start of the next line of treatment, or death, whichever occurred first. Line of treatment was also advanced if there was a gap of greater than 120 days from the end of one treatment regimen to the start of the next regimen.

Clinical outcomes included time to treatment discontinuation (TTD), time to next treatment (TTNT), real‐world (rw) PFS, and rwOS. TTD was defined as the time from treatment initiation of each line of treatment to the earlier of the date of treatment discontinuation or death. TTNT was defined as the time from treatment initiation date to the earlier of the start of first subsequent anticancer treatment or death. These outcomes were assessed from the date of treatment start and by line of treatment. Real‐world progression events in the database were based on findings reported by the treating physician, indicating clinical or radiographic spread of the disease in the physician notes. rwPFS was defined as the time from treatment initiation of each line of therapy to earlier of the date of rw progression events or death. Patients were censored at the date of last follow‐up visit or the end of study (December 31, 2021). rwOS was defined as the time from treatment initiation of each line of therapy to death, with patients censored at the date of the last follow‐up visit or the end of the study.

### 2.4 Analyses

Study variables, including baseline and outcome measures, were assessed descriptively. For categorical variables, counts and percentages were summarized. Means and standard deviations (SD) were reported for continuous variables. Kaplan–Meier analysis, which accounts for variability in the duration of patient follow‐up and censoring, was used to estimate TTNT, TTD, rwPFS, and rwOS for patients with mNSCLC with *HER2* mutations.

## 3 Results

### 3.1 Study Sample

Among 11,889 patients with mNSCLC, 9206 patients had documented HER2 testing results. Of these, 164 patients with *HER2*‐mutant, *EGFR* wild‐type mNSCLC were included in this study. The prevalence of HER2 tumor mutations without EGFR mutations was then estimated to be ~2% (164/9206) (Figure 1). Patient baseline characteristics are shown in Table 1. The majority of patients were women (56.7%), and the mean (SD) age at diagnosis was 67.3 (10.2) years. Most patients were White (63.4%), and 53.0% had a history of smoking. The common sites of metastases at baseline were bone (63.0%), brain (44.0%), liver (29.0%), and lymph nodes (26.0%) (Table 1). The median (quartile 1 [Q1]–quartile 3 [Q3]) follow up was 13.9 (6.9–28.8) months from mNSCLC diagnosis date (Table S2).

**TABLE 1:** Baseline demographic and clinical characteristics.

Characteristics	Cohort with *HER2*‐mutant mNSCLC (*N* = 164)
Female, *n* (%)	93 (56.7)
Age at mNSCLC diagnosis, years, mean (SD)	67.3 (10.2)
Race or ethnicity, *n* (%)	
White	104 (63.4)
Black or African American	10 (6.1)
Asian	9 (5.5)
Hispanic/Latino	0 (0.0)
Unknown/other race	23 (14.0)
Smoking status, *n* (%)	
Former/current smokers	87 (53.0)
Never smokers	77 (47.0)
Unknown/missing status	0 (0.0)
ECOG performance status, *n* (%)	
0–1	112 (68.3)
2–3	22 (13.4)
Unknown	30 (18.3)
Histology, *n* (%)	
Non‐squamous cell carcinoma	158 (96.3)
Squamous cell carcinoma	6 (3.7)
Stage at diagnosis, *n* (%)	
I	> 5 (> 5.5)
II	10 (6.1)
III	18 (11.0)
IV	125 (76.2)
Unknown	< 5 (< 3.0)^a^
Sites of metastasis, *n* (%)	
Bone	104 (63.0)
Brain	72 (44.0)
Liver	48 (29.0)
Lymph nodes	42 (26.0)
Other locations	121 (74.0)

Abbreviations: ECOG, Eastern Cooperative Oncology Group; HER2, human epidermal growth factor receptor 2; mNSCLC, metastatic non‐small cell lung cancer; SD, standard deviation.

^a^ A sample size < 5 was not shown in the table owing to patient privacy.

Median (Q1–Q3) time from mNSCLC diagnosis to NGS testing was 44 days (28–169). Among 164 patients with *HER2* tumor mutations, further details regarding the specific mutations were available for 155 (94.5%) patients. Mutation subgroups included 108 (70%) patients with exon 20 mutations, 24 (15%) with exon 8 mutations, 10 (6.0%) with exon 19 mutations, and the remainder with other HER2 mutations. The most prevalent *HER2* mutation type was exon 20: A775_G776insYVMA (in 44.0% of patients; Table 2).

**TABLE 2:** *HER2* mutation type in cohort of 155 patients^a^ with available mutation details.

*HER2* mutation	*N* (%)
Exon 20: A775_G776insYVMA	68 (44)
Exon 20 G776 > VC	16 (10)
Exon 8 S301F	11 (7.1)
Exon 20 P780_Y781insGSP	10 (6.5)
Exon 8 S335C	7 (4.5)
Exon 8 S310Y	< 5 (< 3.2)^b^
Exon 19 L755P	< 5 (< 3.2)^b^
Exon 20 V777L	< 5 (< 3.2)^b^
Exon 20 G776 > VV	< 5 (< 3.2)^b^
Exon 8 S305C	< 5 (< 3.2)^b^
Exon 17 G660D	< 5 (< 3.2)^b^
Exon 17 V659E	< 5 (< 3.2)^b^
Exon 19 D769Y	< 5 (< 3.2)^b^
Exon 20 G776 > LC	< 5 (< 3.2)^b^
Exon 21 T862A	< 5 (< 3.2)^b^
Exon 3 R103Q	< 5 (< 3.2)^b^
Exon 17 R678Q	< 5 (< 3.2)^b^
Exon 17 S653C	< 5 (< 3.2)^b^
Exon 18 Q709L	< 5 (< 3.2)^b^
Exon 19 D769H	< 5 (< 3.2)^b^
Exon 19 I767M	< 5 (< 3.2)^b^
Exon 19 L755A	< 5 (< 3.2)^b^
Exon 19 L755S	< 5 (< 3.2)^b^
Exon 20 G776_V777 > CVCM	< 5 (< 3.2)^b^
Exon 20 G776C	< 5 (< 3.2)^b^
Exon 20 G776S	< 5 (< 3.2)^b^
Exon 20 G778S	< 5 (< 3.2)^b^
Exon 20 S779Y	< 5 (< 3.2)^b^
Exon 21 V842I	< 5 (< 3.2)^b^
Exon 22 R896C	< 5 (< 3.2)^b^
Exon 24 R970W	< 5 (< 3.2)^b^
Any exon 20 mutations	108 (70)
Any exon 8 mutations	24 (15)
Any exon 19 mutations	10 (6)

Abbreviations: HER2, human epidermal growth factor receptor 2; mNSCLC, metastatic non‐small cell lung cancer.

^a^ HER2 mutation type was not known in 9 of 164 patients with HER2‐mutant mNSCLC.

^b^ Sample size less than 5 was not shown in the table owing to patient privacy.

### 3.2 Treatments

A total of 132 patients received first‐line treatment with the median (Q1–Q3) follow‐up of 13.9 (6.9–28.8) months. The median (Q1–Q3) treatment duration was 9.0 (5–18) months (Table S2). By treatment class (Table S1), platinum‐based chemotherapy (*n* = 60; 45.5%) was the most common treatment in first‐line treatment, followed by ICI with chemotherapy (*n* = 37; 28.0%), ICI monotherapy (*n* = 15; 11.4%), and non‐platinum‐based chemotherapy (*n* = 9; 6.8%) (Table 3). By treatment regimen, the most common treatments were carboplatin/pemetrexed/pembrolizumab (*n* = 32/132; 24.2%), carboplatin/pemetrexed (*n* = 30/132; 22.7%), and carboplatin/pemetrexed/bevacizumab (*n* = 14/132; 10.6%) (Table 4).

**TABLE 3:** Treatments.

Treatment groups, *n* (%)	First‐line treatment (*n* = 132)	Second‐line treatment (*n* = 84)	Third‐line treatment (*n* = 43)
Platinum‐based chemotherapy (± VEGFi or TKI)	60 (45.5)	11 (13.1)	7 (16.3)
ICI (± VEGFi)^a^	15 (11.4)	32 (38.1)	8 (18.6)
ICI + chemotherapy (platinum or non‐platinum based)	37 (28.0)	14 (16.7)	0 (0)
Non‐platinum‐based chemotherapy (± VEGFi)	9 (6.8)	14 (17.0)	14 (32.5)
Trastuzumab‐based regimen	< 5 (< 3.8)^b^	7 (8.3)	9 (20.9)
HER2/EGFR TKI	7 (5.3)	6 (7.1)	< 5 (< 11.6)^b^
VEGFi	< 5 (< 3.8)^b^	< 5 (< 6.0)^b^	< 5 (< 11.6)^b^
Common classes of treatments			
Any chemotherapy (without ICI)	69 (52.3)	25 (29.1)	21 (48.8)
Any ICI (with and without chemotherapy)	52 (39.4)	46 (55.8)	8 (18.6)
Any chemotherapy or ICI	121 (91.7)	71 (84.5)	29 (67.4)

Abbreviations: EGFR, epidermal growth factor receptor; HER2, human epidermal growth factor receptor 2; ICI, immune checkpoint inhibitor; TKI, tyrosine kinase inhibitor; VEGFi, vascular endothelial growth factor inhibitor.

^a^ Alone or in combination with another immunotherapy.

^b^ A sample size of less than 5 was not shown in the table owing to patient privacy.

**TABLE 4:** Most common treatment regimens.

Treatment	*N* (%)
First line of treatment	
Carboplatin, pembrolizumab, pemetrexed	32/132 (24.2)
Carboplatin, pemetrexed	30/132 (22.7)
Bevacizumab, carboplatin, pemetrexed	14/132 (10.6)
Second line of treatment	
Nivolumab	24/84 (28.6)
Atezolizumab	5/84 (6.0)
Third line of treatment	
Ramucirumab + docetaxel	5/43 (11.6)
Docetaxel	5/43 (11.6)

A total of 84/132 (63.6%) received second‐line treatment, and the median (Q1–Q3) treatment duration was 7.0 (3–14) months. The most common treatment by therapy class at second line was ICI‐based therapy (*n* = 32/84; 38.0%), followed by ICI + chemotherapy (*n* = 14/84; 17.0%), non‐platinum‐based chemotherapy (*n* = 14/84; 17.0%), and platinum‐based chemotherapy (*n* = 11/84; 13.0%) (Table 3). By treatment regimen, the most frequently used treatments were nivolumab (*n* = 24/84; 28.6%) and atezolizumab (*n* = 5/84; 6.0%) (Table 4).

A total of 43/132 (32.6%) patients had progressed to third‐line of treatment and the median (Q1–Q3) duration of treatment was 8.0 (3–15) months (Table S2). Non‐platinum‐based chemotherapy (*n* = 14/43; 33.0%) was the most frequently used treatment in third line, followed by trastuzumab‐based regimens (*n* = 9/43; 21.0%), ICI‐based therapy (*n* = 8/43; 19.0%), and platinum‐based therapy (*n* = 7/43; 16.0%) (Table 3). By treatment regimen, the most common treatments were ramucirumab/docetaxel (*n* = 5/43; 11.6%) and docetaxel (*n* = 5/43; 11.6%) (Table 4).

More than 60% of patients were treated with platinum‐based or non‐platinum‐based chemotherapy, ICI, or chemotherapy + ICI across all three lines of treatments. These regimen types were received by 91.7% (first‐line treatment), 84.5% (second‐line treatment), and 67.4% (third‐line treatment) of patients. The predominant use of platinum‐based chemotherapy (45.5%) and ICI with or without chemotherapy (39.4%) was observed in first‐line of treatment, whereas the proportions of patients in this treatment category diminished in second and third line of treatment (Table 3).

For second and third line of treatment, 7/84 (8.3%) and 9/43 (20.9%) patients received trastuzumab‐based regimens, respectively (number of patients in first‐line treatment not reported owing to small sample size). The observed HER2‐directed treatments included trastuzumab, T‐DM1, or T‐DXd either as monotherapy or in combination with chemotherapy. T‐DXd use was off label during the study period.

### 3.3 Clinical Outcomes

Median TTD was 4.2 (95% CI 3.5, 4.5) months, 4.2 (95% CI 3.0, 6.2) months, and 5.2 (95% CI 2.9, 7.7) months for first, second, and third line of treatment, respectively. Median TTNT was 7.1 (95% CI 5.7, 8.9) months, 4.8 (5% CI 3.8, 7.3), and 5.2 (95% CI 3.0, 8.7) months for first, second, and third line of treatment, respectively (Table S3).

**FIGURE 2:**
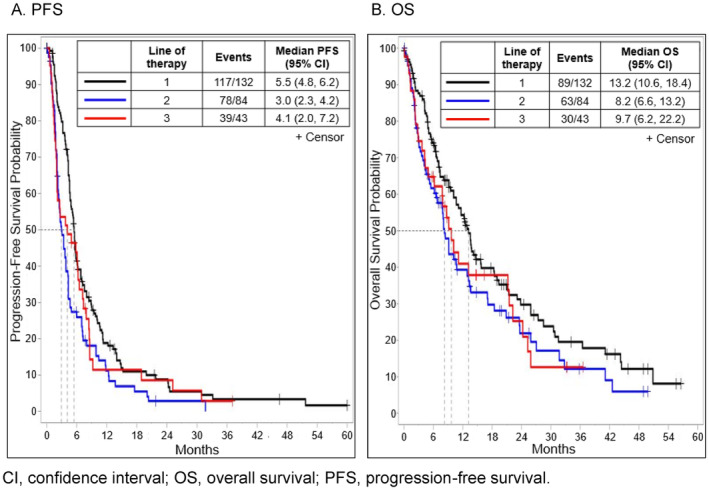
Kaplan–Meier plots for PFS (A) and OS (B) by line of therapy. CI, confidence interval; OS, overall survival; PFS, progression‐free survival.

Based on disease progression assessed by healthcare providers, median rwPFS across all treatments was 5.5 (95% CI 4.8, 6.2) months, 3.0 (95% CI 2.3, 4.2) months, and 4.1 (95% CI 2.0, 7.2) months in first, second, and third line of treatment, respectively. Median rwOS in first line of treatment combining all treatments was 13.2 (95% CI 10.6, 18.4) months. Similar to median rwPFS, median rwOS also became shorter in later lines of treatment. Median rwOS in second and third line of treatment was 8.2 months (95% CI 6.6, 13.2) and 9.7 months (95% CI 6.2, 22.2), respectively (Figure 2).

## 4 Discussion

In this real‐world study of data from patients with *HER2*‐mutant mNSCLC without evidence of *EGFR* mutations, using combined EHRs with comprehensive genomic analysis, we described the prevalence of *HER2* mutations among patients with mNSCLC, characterized patients with *HER2*‐mutant mNSCLC, and outlined treatments and associated clinical outcomes. The prevalence of *HER2*‐mutant mNSCLC without concurrent *EGFR* mutations was 2% among patients who received tissue NGS testing, consistent with previous studies reporting a prevalence of 2%–4% [13, 34].

Patients with *HER2*‐mutant mNSCLC have previously been described as predominantly female and never smokers [15]. In our study, patients tended to be female (56.7%), and about half of them had no history of smoking (47.0%). The mean age of patients was 67.3 years. Our findings are similar to those of prior real‐world studies, which reported median ages of 61–66 years and 32%–76% with non‐smoking history [13, 17, 35, 36, 37]. *HER2*‐mutant NSCLC is additionally related to a higher proportion of brain metastases than NSCLC with other mutations. In our study, 44.0% of patients had brain metastasis at baseline, similar to findings from a prior study detecting a greater percentage of brain metastases with *HER2*‐mutant mNSCLC compared with mNSCLC driven by other mutations such as *KRAS* [38].

The study findings demonstrated significant variability in the types of treatments received in the second‐ and third‐line settings for *HER2*‐mutant mNSCLC. Whereas platinum‐based chemotherapy was received by almost half of the patients in the first‐line setting, there was no predominant treatment choice in second and third line. ICI was used in 38% of patients in second line and non‐platinum‐based chemotherapy in 33% of patients in third line. The lack of standard of care was more evident at the treatment regimen level in second and third line, where substantial variability was observed. These findings suggest a need for more effective and standardized treatment options after patients progress on platinum‐based chemotherapy in the first‐line setting.

This study reported treatment patterns before the approval of a targeted treatment for *HER2*‐mutant mNSCLC to provide a historical context of the treatment landscape that has since evolved. Treatments used were similar to what would be expected for patients with mNSCLC with no targetable mutations. Chemotherapy or IO (± chemotherapy) was the most frequently used treatment during the period study for all three lines of treatment, with carboplatin/pemetrexed ± pembrolizumab being the most prevalent regimen in the first‐line setting. This observation is also consistent with the National Comprehensive Cancer Network (NCCN) guidelines during the study period prior to the approval of HER2‐directed therapy where ICI ± chemotherapy was recommended for mNSCLC without a driver mutation [4]. Several retrospective studies reported that ICI monotherapy may have limited clinical benefits in HER2‐mutant mNSCLC [39, 40, 41]. For ICI + chemotherapy option, there is a lack of data comparing the efficacy of HER2‐directed therapy vs. ICI + chemotherapy in this patient population in first line setting. The ongoing DESTINY‐04 trial is investigating this research question to guide treatment decisions [42]. Only a small number of patients with *HER2* tumor mutations received HER2‐directed therapies in our study, although the proportion of patients who received HER2‐directed therapy increased in later lines of treatment (21% in third line). The lack of use of HER2‐directed therapies can be explained by the fact that there were no approved HER2‐directed treatments for mNSCLC during the study period. This result may also indicate that HER2‐directed treatments were more likely to be considered in later lines of treatment after chemotherapy and ICI treatment options are exhausted owing to the lack of approved HER2‐directed treatments. Another finding to note is that trastuzumab with chemotherapy was used in all three lines of treatment (used in fewer than five patients in each line of treatment), although this treatment combination has never been recommended by the NCCN guidelines and data are lacking from clinical trials to support its use [4, 26, 43].

Several real‐world studies have been published on the treatment patterns and clinical outcomes in HER2‐mutant mNSCLC in the last 10 years [13, 17, 35, 36, 37, 44]. One of these studies was conducted in the US and included a contemporary cohort of patients similar to our study. That retrospective, single‐institution study by Waliany et al. at an academic center in the US assessed outcomes in patients with *HER2*‐mutant/*EGFR* wild‐type mNSCLC (*n* = 33) identified from 2012 to 2021. That study reported similar first‐line treatment patterns as our study, with the most common first‐line treatments being platinum‐based chemotherapy (67.7%), chemotherapy with ICI (16.1%), and ICI monotherapy (6.5%). There was, however, more frequent use of platinum‐based chemotherapy (67.0% vs. 45.0%) and less frequent use of ICI (22.6% vs. 36.0%) compared with our findings with the FH‐FMI CGDB. In addition, the use of HER2‐directed treatments in the study by Waliany et al. was much higher in second line (52.0%) than in our study (8.3%). Despite the similar study periods, these differences in the use of HER2‐directed treatments may be attributable to our data coming mostly from community oncology practices (88.0%), reflecting variable practice patterns in the community relative to academic centers. Although the sample size was small, Waliany et al. reported the highest response rates with T‐DXd (ORR 40.0%) compared with other HER2‐directed therapies (7.1% for trastuzumab/chemotherapy combination and 0% for T‐DM1) [35].

In other retrospective studies at academic centers both in and outside of the US, a wide range of OS rates for *HER2*‐mutant mNSCLC have been reported, from 11.5 to 24.0 months. These studies were notable for significant variability in the proportion of patients treated with HER2‐directed therapies (ranging from 10.0% to 64.0%) and in the types of HER2‐directed therapies received. The median OS in our study falls within the range reported from other real‐world studies with median rwOS of 13.2 months from first‐line treatment initiation among patients who received care mostly from community oncology practices (88.0%) [13, 17, 35, 36, 37, 44]. More data assessing the efficacy of HER2‐directed therapies in real‐world practice, especially in community‐based oncology practices, are needed.

In clinical trials, data on the efficacy of HER2‐directed ADCs have been favorable for T‐DXd and with less established efficacy for T‐DM1. A Phase 2 basket trial assessing T‐DM1 in *HER2*‐mutant mNSCLC demonstrated an ORR of 44.0% and an mPFS of 5 months, resulting in NCCN recommendation of T‐DM1 [45]. Other T‐DM1 studies, however, showed inconsistent results. Another Phase 2 trial of T‐DM1 in NSCLC with *HER2* alterations (*n* = 7 for *HER2* mutations) ended early owing to limited efficacy with an ORR of 6.7% and an mPFS of 2.0 months [22, 45]. A third study of T‐DM1 in patients with mostly *HER2* exon 20 mutations (*n* = 22) reported an ORR of 38.1% with an mPFS of 2.8 months [23]. Regarding T‐DXd, data have been reported in two Phase 2 trials in patients with previously treated *HER2*‐mutant NSCLC. In DESTINY‐Lung01, T‐DXd 6.4 mg/kg showed an ORR of 55% and an mPFS of 8.2 months [15]. T‐DXd (5.4 mg/kg) in the DESTINY‐Lung02 study demonstrated an ORR of 57.7% (49%) and a duration of response of 8.7 (16.8) months (16.8), with a mPFS by blinded independent central review of 9.9 months (95% CI 7.4, NE) and a mOS of 19.5 months (95% CI 13.6, NE) [24, 46]. In our study, median rwPFS decreased in later lines of treatments and was less than 5 months following second‐ and third‐line treatments. Similarly, median rwOS was less than 9 months in patients who received second‐ and third‐line treatments. Although not a head‐to‐head comparison, the contrast between these findings suggests that T‐DXd leads to better survival outcomes compared with other therapies in patients who progressed after first‐line treatment for *HER2*‐mutant mNSCLC. Given the recent approval of T‐DXd in previously treated mNSCLC, the need to perform tumor NGS, including *HER2* mutation testing, for patients with NSCLC should be emphasized in routine clinical care [4].

There are several limitations to our study. The study was descriptive in nature, with small sample sizes in each treatment category. In addition, the patient identification period (ending July 2021) may have limited the sample size of patients who received the only approved HER2‐directed therapy since T‐DXd obtained the FDA approval in August 2022. Therefore, we could not compare outcomes between treatment categories, especially among HER2‐directed therapies. HER2 expression by IHC score was also not available for assessment in our study. Hence, our data cannot be extrapolated to the more recent indication for T‐Dxd in advanced NSCLC with HER2 expression detected by IHC. The follow‐up in the study may not have been long enough to assess survival events in some patients who were censored. The clinical outcomes need to be interpreted in that context. Future studies should evaluate efficacy and the optimal sequencing of treatments with ADCs, HER2/EGFR TKIs, and emerging new TKIs for their impact on subsequent patient outcomes, and potentially even consider the combination of these agents with other anticancer therapies.

## 5 Conclusions

Before the approval of T‐DXd, treatments in mNSCLC with *HER2* mutations were predominantly chemotherapy or ICI, similar to what would generally be given to patients whose tumors have no actionable mutations. Median rwOS was 13.2 months and 8.2 months from first‐ and second‐line treatment initiation, respectively, in this study, where most patients received platinum‐based chemotherapy ±IO. These results indicate the need for more effective first‐line treatment options for *HER2*‐mutant mNSCLC. With the recent approval of HER2‐directed therapy such as T‐DXd in previously treated *HER2*‐mutant mNSCLC, routine testing of *HER2* mutations in NSCLC should be incorporated into clinical practice. Future studies are also warranted to assess potential changes in treatment patterns and associated outcomes.

## Author Contributions

**Sarah Waliany:** conceptualization (equal), investigation (lead), methodology (lead), supervision (equal), writing – original draft (lead), writing – review and editing (lead). **Misako Nagasaka:** conceptualization (equal), investigation (lead), methodology (lead), supervision (equal), writing – original draft (equal), writing – review and editing (equal). **Leah Park:** conceptualization (equal), formal analysis (equal), investigation (equal), project administration (equal), supervision (equal), writing – original draft (lead), writing – review and editing (lead). **Clara Lam:** conceptualization (equal), investigation (equal), methodology (equal), writing – original draft (supporting), writing – review and editing (supporting). **Zoe Jiang:** data curation (lead), formal analysis (lead), methodology (supporting), software (lead), validation (lead), visualization (supporting), writing – review and editing (supporting). **Feng Lin:** conceptualization (equal), investigation (supporting), methodology (supporting), writing – original draft (supporting), writing – review and editing (supporting). **Joel W. Neal:** conceptualization (equal), formal analysis (equal), methodology (equal), supervision (lead), writing – original draft (equal), writing – review and editing (equal).

## Conflicts of Interest

Dr. Waliany received consulting fees from AstraZeneca. Dr. Nagasaka reports receiving consulting fees from Caris Life Sciences, honoraria from AstraZeneca, Daiichi Sankyo, Novartis, Eli Lilly, Pfizer, EMD Serono, Genentech, Regeneron and BMS, speaker for Mirati, Takeda, Janssen, and Blueprint Medicine and travel support from AnHeart Therapeutics. Dr. Park and Dr. Lam are employees of AstraZeneca. Ms. Jiang was an employee of AstraZeneca at the time of the study. Dr. Lin was an employee of Daiichi Sankyo at the time of the study. Dr. Neal reports receiving consulting fees from CME Matters, Clinical Care Options CME, Research to Practice CME, Medscape CME, Biomedical Learning Institute CME, MLI PeerView CME, Projects in Knowledge CME, Rockpointe CME, MJH Life Sciences CME, Medical Education Consortium, and HMP Education, consulting or advisory role to AstraZeneca, Genentech/Roche, Exelixis, Takeda Pharmaceuticals, Eli Lilly and Company, Amgen, Iovance Biotherapeutics, Blueprint Medicines, Regeneron Pharmaceuticals, Natera, Sanofi, D2G Oncology, Surface Oncology, Turning Point Therapeutics, Mirati Therapeutics, Gilead Sciences, Abbvie, Summit Therapeutics, Novartis, Novocure, Janssen Oncology, and AnHeart Therapeutics and research funding from Genentech/Roche, Merck, Novartis, Boehringer Ingelheim, Exelixis, Nektar Therapeutics, Takeda Pharmaceuticals, Adaptimmune, GSK, Janssen, Abbvie, and Novocure.

## Supporting information


Table S1.
Table S2.
Table S3.
Table S4.

## Data Availability

The data originated from Flatiron Health and Foundation Medicine Inc. Requests for data sharing by license or by permission for the specific purpose of replicating results in this manuscript can be made to publicationsdataaccess@flatiron.com and cgdb‐fmi@flatiron.com.
