# Supplementary material for: Real‐World Prevalence, Treatment Patterns, and Outcomes for Patients With HER2 (ERBB2)‐Mutant Metastatic Non‐Small Cell Lung Cancer, From a US‐Based Clinico‐Genomic Database
Source: Cancer Med. 2024 Dec 18;13(24):e70272. doi: 10.1002/cam4.70272 (PMC11653943; doi:10.1002/cam4.70272)
Supplement: Supplementary file 1 — Table S1. Table S2. Table S3. Table S4. [file CAM4-13-e70272-s001.docx]

**Supplementary Table 1. Treatment regimens received stratified by therapy class**

| **Therapy class** | **Regimen** |
| --- | --- |
| HER2/EGFR TKI | Afatinib |
| ICI ± VEGFi | Atezolizumab |
|  | Atezolizumab, bevacizumab |
|  | Ipilimumab, nivolumab |
|  | Nivolumab |
|  | Pembrolizumab |
| ICI + chemotherapy | Carboplatin, paclitaxel protein bound, pembrolizumab |
|  | Carboplatin, paclitaxel, pembrolizumab |
|  | Carboplatin, pembrolizumab, pemetrexed |
|  | Pembrolizumab, pemetrexed |
| Non-platinum-based chemotherapy (± VEGFi) | Bevacizumab, pemetrexed |
|  | Docetaxel |
|  | Docetaxel, ramucirumab |
|  | Fluorouracil, irinotecan, leucovorin |
|  | Gemcitabine |
|  | Gemcitabine, vinorelbine |
|  | Paclitaxel |
|  | Paclitaxel protein bound |
|  | Pemetrexed |
|  | Vinorelbine |
| Platinum-based chemotherapy (± VEGFi or TKI) | Bevacizumab-awwb, carboplatin, paclitaxel |
|  | Bevacizumab-awwb, carboplatin, pemetrexed |
|  | Bevacizumab-bvzr, carboplatin, pemetrexed |
|  | Bevacizumab, carboplatin, docetaxel, pemetrexed |
|  | Bevacizumab, carboplatin, gemcitabine, paclitaxel protein bound, pemetrexed |
|  | Bevacizumab, carboplatin, paclitaxel |
|  | Bevacizumab, carboplatin, pemetrexed |
|  | Carboplatin, etoposide |
|  | Carboplatin, gemcitabine |
|  | Carboplatin, paclitaxel |
|  | Carboplatin, paclitaxel protein bound |
|  | Carboplatin, pemetrexed |
|  | Cisplatin, etoposide |
|  | Cisplatin, paclitaxel |
|  | Cisplatin, paclitaxel protein bound |
| Trastuzumab-based therapy | Ado-trastuzumab emtansine |
|  | Ado-trastuzumab emtansine, afatinib |
|  | Bevacizumab, carboplatin, paclitaxel, trastuzumab |
|  | Carboplatin, paclitaxel, trastuzumab |
|  | Docetaxel, pertuzumab, ramucirumab, trastuzumab |
|  | Docetaxel, trastuzumab |
|  | Fam-trastuzumab deruxtecan-nxki |
|  | Paclitaxel protein bound, trastuzumab |
|  | Paclitaxel protein bound, trastuzumab-anns |
| VEGFi | Bevacizumab-bvzr |

EGFR, epidermal growth factor receptor; HER2, human epidermal growth factor receptor 2; ICI, immune checkpoint inhibitor; TKI, tyrosine kinase inhibitor; VEGFi, vascular endothelial growth factor inhibitor.

**Supplementary Table 2. Number of lines of treatment and follow-up period**

| Total number of lines of treatment received from mNSCLC diagnosis to end of follow up, n (%)  0  1  2  3  4  5+ | 32 (19.5)  48 (29.3)  41 (25.0)  18 (11.0)  18 (11.0)  7 (4.3) |
| --- | --- |
| Duration of follow up from mNSCLC diagnosis,  median (Q1–Q3), months | 13.9 (6.9–28.8) |
| Duration of follow up from line of treatment initiation,  median (Q1–Q3), months  First line (n=132)  Second line (n=84)  Third line (n=43) | 9 (5–18)  7 (3–14)  8 (3–15) |
| Time from mNSCLC diagnosis to first NGS testing result, median (Q1–Q3), months | 44 (28–169) |

mNSCLC, metastatic non-small cell lung cancer; NGS, next-generation sequencing; Q1, quartile 1; Q3, quartile 3.

**Supplementary Table 3. Treatment outcomes**

| **Outcome** | **First-line treatment**  **(n=132)** | **Second-line treatment**  **(n=84)** | **Third-line treatment**  **(n=43)** |
| --- | --- | --- | --- |
| TTD, median (95% CI), months | 4.2 (3.5, 4.5) | 4.2 (3.0, 6.2) | 5.2 (2.9, 7.7) |
| TTNT, median (95% CI), months | 7.1 (5.7, 8.9) | 4.8 (3.8, 7.3) | 5.2 (3.0, 8.7) |

CI, confidence interval; TTD, time to treatment discontinuation; TTNT, time to next treatment.
